# The Prevalence of Domestic Violence in the Lives of Female Heterosexual Partners of Sex Addicts

**DOI:** 10.1177/10778012231199111

**Published:** 2023-09-12

**Authors:** Lisa Taylor, Irene De Haan

**Affiliations:** Department of Counselling, Human Services and Social Work, 183465 University of Auckland, Auckland, New Zealand

**Keywords:** partner of sex addict, domestic violence, sex addiction, problematic pornography use, intimate partner violence

## Abstract

This study provides evidence that rates of domestic violence (DV) run considerably higher in the lives of heterosexual women who identify as partners of sex addicts (PSAs) than in the general population. Data collected from 558 survey participants, from a variety of high-income nations, revealed that 92.1% had ever experienced any form of DV perpetrated by their partner and 57.7% had experienced physical and/or sexual intimate partner violence with their partner. The study also tests several hypotheses about sex addiction behaviors and PSA intimate partner violence (IPV), to help those working with these populations understand what factors may be contributing to, or mitigating, these women's experiences of violence.

Domestic violence (DV) features in the lives of numerous women and children around the world. This study sheds light on the prevalence and frequency of various forms of DV—emotional, physical, sexual, coercive control, and abuse of children—in the lives of a subset of women who identify themselves as heterosexual partners of sex addicts (PSAs). In addition to determining violence prevalence and frequency, the current study explores associations between violence and factors such as PSA age, length of time in the relationship, specific problematic sexual behaviors (PSBs) her partner has participated in, and also his recovery behaviors. Finally, the study provides information on help-seeking behaviors that participants have engaged in and found beneficial.

Researchers of violence against women (VAW; Ellsberg et al., 2003; Fanslow & Robinson, 2011) have noted that measurement of DV is important in that it enables mental health service providers, and other helping professionals, to provide better care for individuals who are affected, or who are likely to be affected, and “for informing intervention and prevention efforts” (Fanslow & Robinson, 2011, p. 742). Frequency data regarding DV is useful as frequency of the violence is considered by researchers to be one of the key elements contributing to DV trauma (Sanderson, 2008; Warshaw & Sullivan, 2013).

The sex addiction or PSB counseling field has long acknowledged that sexual disorders are “frequently associated with DV” (Irons & Schneider, 1997, p. 341); however, prior to this study, there has been no specific research on this population to back up this observation. There have, however, been studies that come close, particularly the work of VAW researchers—often feminist academics (Dines & Jensen, 2008; Russell, 1998) and critical criminologists (DeKeseredy, 2015; Hall-Sanchez, 2014). These researchers, however, have often focused on the *pornography* use of violence perpetrators. A small handful of them have looked at the perpetrator's use of the sex industry (Farley et al., 2017; Simmons et al., 2008), or other types of “impersonal” sexual acting out behaviors (Malamuth et al., 1995). None of the aforementioned researchers has, however, applied an addictions or PSB paradigm to the sexual acting out. That said, in a few recent studies (Borgogna et al., 2020; Brem et al., 2018) a DV perpetrator's pornography use has been categorized as “problematic” using a standardized assessment tool (Kor et al., 2014).

There are, however, DV studies which focus on the role of other types of addictions in the violence. For example, there exists a large, international body of research on the influence of substance addiction on intimate partner violence (IPV) or DV (Hussain et al., 2020; Shorey et al., 2018). The significant differences between sex addiction (a “process addiction,” like gambling and food addiction) and substance, or chemical addictions, however, render these studies somewhat less useful to the sex addiction/betrayal trauma counselor than the aforementioned VAW studies. That said, there also exists research demonstrating an association between the process addiction, gambling, and violence against the addict's intimate partner (Muelleman et al., 2002) and children (Kalischuk et al., 2006).

And while these studies are certainly helpful to the sexual addiction/betrayal trauma therapeutic community, there seemed to be a place for a study which would quantify how prevalent and frequent various forms of DV have been in the lives of PSAs. To begin, however, it would be helpful to consider the development of the concepts of sex addiction/PSB, and PSA betrayal trauma.

## Development of the Concept of “Sex Addiction”

In Western medicine, excessive, impersonal, sexual behaviors were documented in the eighteenth, nineteenth, and early twentieth centuries by various European and American doctors and sexologists (Kafka, 2010). These writings were precursors to the later twentieth-century characterization of habitual promiscuity as, “Don Juanism” or “satyriasis” in males and “nymphomania” in females (Kafka, 2010). These terms would make their way into the DSM-III in the United States and ICD-9, along with categories for paraphilic behaviors (Kafka, 2010). “Nymphomania” and “Don Juanism” would also become part of the common vernacular.

By the mid-80s researchers working from an addictions model began to label obsessive and/or compulsive impersonal sexual acting out as “sex addiction” (Carnes, 2001). This genderless term gradually replaced earlier terms, and the model that Patrick Carnes introduced for the treatment of this problem—including his “30 tasks of sex addiction recovery” model—came, in time, to the fore in the therapeutic community (Carnes, 2001).

As the new millennium has progressed, other models and other terms have arisen. These terms—including “sexual compulsivity” (Society for the Advancement of Sexual Health, 2018), “hypersexual disorder” (Samenow, 2010), “Compulsive Sexual Behavior Disorder” (WHO, 2019), “Chronically Problematic Sexual Behavior” (Herring, 2017), and “Compulsive-Abusive Sexual-Relational Disorder” (CASRD; Minwalla, 2021)—come from a variety of professional fields including addictions counseling, psychiatry, and psychology. As regards the new models, most acknowledge the contribution of Carnes and his “sex addiction” framework to the field, but seek to achieve other goals, such as medicalizing the term for diagnostic purposes, making space for an etiology besides addiction, or acknowledging the relational abuses that so commonly accompany these sexual behaviors.

The working definition for this study of “sex addiction,” or “PSB,” as it has become increasingly known, is “an obsessive relationship to sexual thoughts, fantasies or activities that an individual continues to engage in despite adverse consequences” (PsychCentral, n.d.f). The person with the PSB will frequently be referred to, for the sake of simplicity, by the acronym “SA.”

## Betrayal Trauma and PSAs

Toward the new millennium, a diversification in couple counseling began, so that it became more inclusive to multicultural and feminist perspectives (Gurman, 2015, p. 6). As part of this broadening of perspectives, female theorists have given us new treatment frameworks to use with individuals and couples facing infidelity. The results have been the investment model (Rusbult et al., 1998), emotionally focused couple therapy (Johnson et al., 1999), and the multidimensional partner trauma model (M-PTM; Association of Partners of Sex Addicts Trauma Specialists (APSATS), 2017).

The latter two models view intimate partner infidelity as an adult-attachment injury (Johnson et al., 1999; Steffens & Rennie, 2006). Thus, rather than seeing a PSA's, or betrayed wife's, strongly emotional responses to betrayal as “maladaptive” (Carnes, 2001), Steffens and Rennie asked therapists to consider that the “symptoms (rage, hyper-vigilance, etc.)… are common responses to significant life-altering traumatic events” (2006, p. 248). They concluded their article with a discussion of the benefits of using a trauma model to help wives of SAs find healing.

While originally coined by psychologist Jennifer Freyd in 1991 to describe childhood sexual abuse (Vogeler et al., 2020), the term “betrayal trauma” would be expanded, in the second decade of the new millennium, to encompass trauma from “sexual infidelity” (Freyd & Birrell, 2013). This inclusion would help to further acceptance in the therapeutic community of the concept of PSA betrayal trauma and in 2017 the Trauma Inventory for PSAs (TIPSA; Vogeler et al., 2018) was developed and tested.

Prior to moving into the field of PSA betrayal trauma, Steffens worked in the DV field with survivors of many types of interpersonal violence and abuse (B. Steffens, personal communication, August 10, 2021). The M-PTM (APSATS, 2017), which was born out of her research and is still taught today by APSATS, trains those working with PSAs to assess for relational abuses, including DV. APSATS also advocates for therapies that do not discriminate against women or blame victims of violence or betrayal (2017).

## Method

Quantitative methods were chosen for this study as such research is useful to “test hypotheses that we have about people, their attitudes or their behaviors” (Williams, 2018, p. 20). The technique for gathering the study data was an online, anonymous, self-report survey comprised of 16 questions. The measures of this survey can be broken into four major groups: (a) standard and PSA/SA-specific demographic measures, (b) the World Health Organization's (WHO's) VAW measures, (c) measures on violence against children, and (d) measures of help-seeking-behavior.

Demographic data were collected to allow the researchers to explore whether any association exists between those participants experiencing physical and/or sexual intimate partner violence (P/SIPV) and factors such as participant age, geographic location, length of time in relationship, etc. The reason for exploring the association between the demographic data and P/SIPV data only, and not the entire DV dataset, was that this allowed for more accurate comparison of the results with WHO's (2013) P/SIPV and other international IPV study results.

The study's VAW measures were based on the WHO “Multi-Country Study on Women's Health and Domestic Violence Against Women” measures 703–706 (Garcia-Moreno et al., 2005). These measures were adapted slightly to adjust for the fact that all participants of this study were being asked to answer for their relationship with their “current or most recent SA husband/partner” rather than their “current or most recent husband/partner, or any other partner” (Garcia-Moreno et al., 2005, p. 136). Moreover, regarding the question on “choking,” the researchers decided to expand the measure to include any behavior restricting breathing, for example, strangulation, attempted drowning, using a body part other than hands to put pressure on the esophagus. Other than this, the measures are the same.

The next DV measure was on violence against a PSA's children by her husband/partner with the sexual addiction. This researcher-devised measure follows the style of the WHO VAW measures: a “yes/no” question related to an act of violence followed by a question on whether the violence is past or current. WHO measures current violence by a “yes/no” question on whether the act was committed within the last 12 months. Finally, the participant is asked about the frequency of the violence (whether current or past) using a graduated scale: *once*, *a few times*, *many times*.

The last measure of the survey was created to explore a topic on which IPV researchers frequently want more data: what, if any, helping-seeking behaviors have the participants engaged in (Fanslow & Robinson, 2010). Participants who had identified that they or their children had experienced acts of violence at the hands of the SA partner were asked if they had ever told anyone about the violence. Those who said they had were asked to identify who they had told: choosing from a list representing various personal relationships and helping professionals. Finally, they were asked who amongst these they had found helpful to tell.

### Participants

Participants for this study were recruited directly (PSA-focused social media, websites, mailing lists), indirectly (colleague mailing lists), and through snowballing (PSAs encouraged to share with other PSAs). Inclusion criteria for participants were English literacy; an age of 18 + years; cohabitation with the person with the addictive behaviors (or separation within the last 24 months); self-identification as a female, heterosexual, PSA; and having sought assistance specifically as the partner of a sex addict.

### Analysis

Three major types of analyses were conducted for this study, one set for each of the three study aims. The first was analysis of prevalence of DV and P/SIPV in the lives of study participants based on descriptive statistics. The second analysis was a comparison of this statistical data against the WHO general population DV data worldwide and in “high-income” countries, which included stratifying some of the demographic data and seeing if the same patterns would emerge (e.g., with regards to participant age and violence). The third set of analyses was conducted to discover what, if any, correlations could be seen to exist between the DV being experienced by participants and various behavioral factors, such as the SA partner's acting out behaviors and engagement in recovery processes. These analyses were made by testing some of the researcher's hypotheses about such behaviors and the existence of historical violence or current violence.

### Data Analysis

Survey data was collected in Qualtrics over the course of 6 months: August 1, 2020 to February 12, 2021. Violence statistics were calculated after creating new variables for combinations of the various classes of violence (e.g., “combined control” was the number of participants reporting “yes” to any type of control). By this means the prevalence of all the various types of violence reported by participants was arrived at, as well as information on frequency and whether the violence was historical or current.

Next, the researchers looked at data from other DV studies, including the WHO's (2013), “Global and Regional Estimates of Violence against Women” report—which is itself a systematic review of data on IPV—to see how the experiences of this population of PSAs compared with global, and high-income populations of women. The researchers also decided to follow the example of Garcia-Moreno et al. (2005) and stratify all categories of physical and sexual violence by the demographic variables “participant age” (see Table 1) and “length of years in the relationship with the SA spouse/partner” to see what trends emerged.

**Table 1.** Participant Age and Physical/Sexual IPV.

Age	Physical violence		Sexual violence		Physical and/or sexual violence		***n***
	Ever (%)	Current (%)	Ever (%)	Current (%)	Ever (%)	Current (%)	
18–19	0.0	0.0	0.0	0.0	0.0	0.0	1
20–24	66.7	33.3	33.3	33.3	66.7	66.7	3
25–29	31.3	25.0	25.0	18.8	50.0	43.8	16
30–34	15.0	15.0	45.0	27.5	50.0	32.5	40
35–39	39.1	23.0	36.8	14.9	58.6	32.2	87
40–44	28.6	12.9	38.6	18.6	50.0	25.7	70
45–49	43.7	19.7	46.5	12.7	64.8	25.4	71
50–54	43.6	23.4	43.6	12.8	62.8	28.7	94
55–59	33.3	11.1	44.4	7.8	64.4	16.7	90
60–64	29.4	11.8	43.1	13.7	47.1	23.5	51
65–69	25.0	15.0	35.0	20.0	50.0	25.0	20
70+	53.3	13.3	40.0	6.7	60.0	13.3	15
All women	35.3	17.4	41.4	14.5	57.7	26.3	558

Finally, various statistical analyses were run on the data for purposes of hypothesis testing (Berman & Wang, 2017). One of the first analyses was creating a Chi-square matrix in SPSS which examined the association between the various forms of SA acting out behaviors and various types of P/SIPV. To facilitate the description of these results, combined variables for the behaviors (e.g., “illegal behaviors,” “acting out with other people”) were also created and Chi-square analysis was performed on these and the various individual and combined P/SIPV variables.

## Results

The current study sought to understand whether DV is being commonly experienced by heterosexual women seeking support for the impact of their partner's sex addiction. According to the study results, the answer is “yes, it is highly prevalent” with (figures rounded) 92% experiencing DV ever, 80% currently; 58% experiencing sexual and/or physical violence ever, 26% currently. Moreover, the findings show that a large proportion of the violence experienced is severe and frequent.

### Participant Demographics

Of the 724 people who engaged with the study, 558 completed the survey in its entirety. These 558 participants were recruited across multiple English-speaking countries; however, the vast majority of them were residing in the United States (79.6%) at the time they completed the study. Most of the rest of the participants were in other English-speaking countries. A few individual participants were residing in non-English-speaking countries.

### Relationships

Four hundred nineteen of the participants (75.1%) were currently still cohabiting with their partner with the PSBs at the time of participation in the study. When asked how long they had been in relationship with their spouse/partner, the most commonly chosen response was 30+ years (25.6%). Regarding children, 88.4% of the participants responded “yes” to the question: “do you have, or have you had, children?” and 58.1% said they are currently living with a child or children.

### Person With the PSBs

When asked about the help-seeking behaviors of their SA spouse or partner, 87.1% of the women said their partner had sought help, with 75.4% reporting their spouse had sought out “specialized professional support for this issue.” Participants were also asked to identify all the sexually addictive behaviors they were aware their partner/spouse had engaged in. The most reported behavior (94.6%) was internet pornography use. Table 2 includes a list of all the PSBs participants were asked to report on.

**Table 2.** Chi-Square Matrix: Acting Out Behaviors and Current Violence.

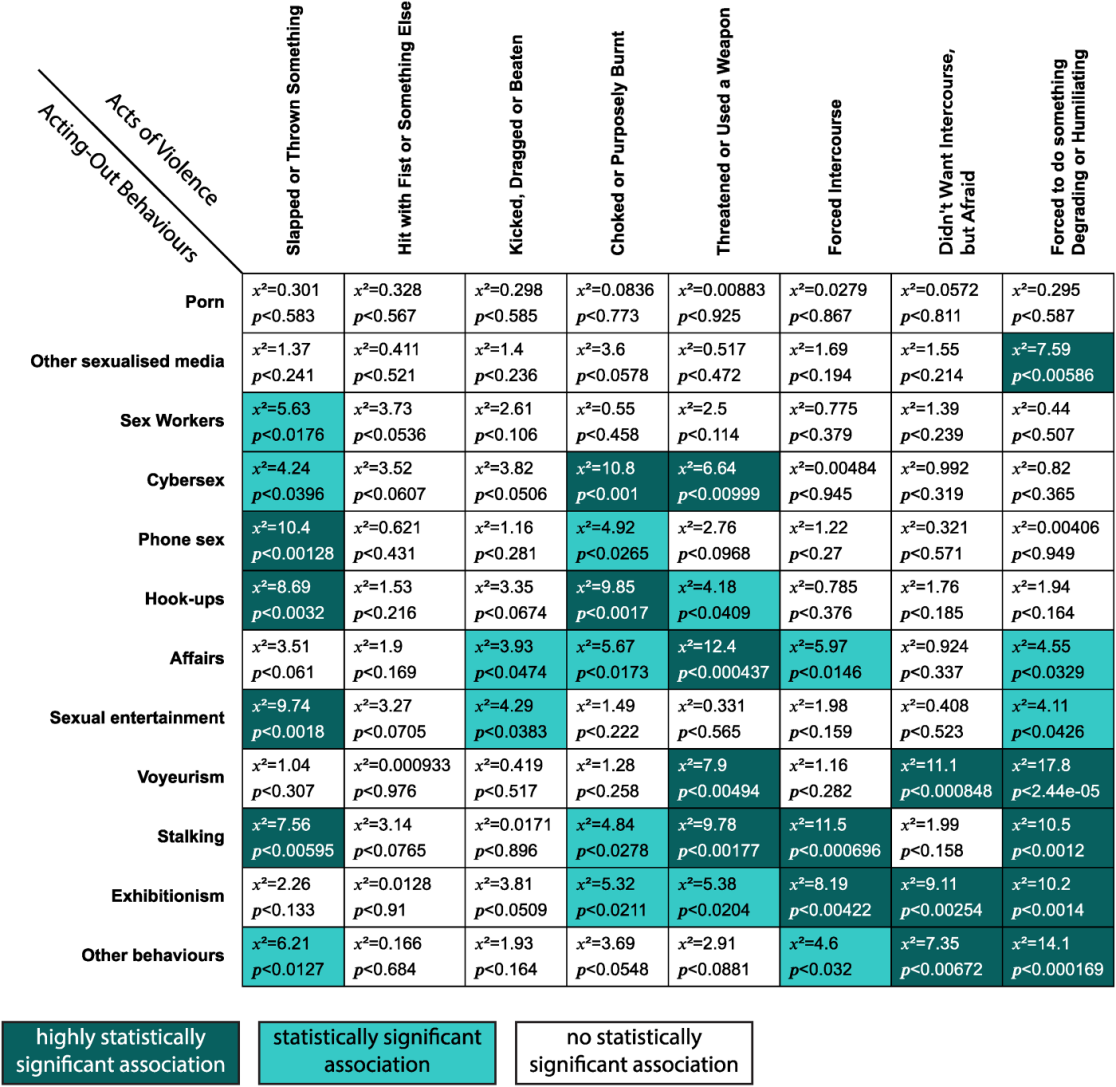

### Experiences of Violence

#### Control

The measure for coercive control, resulted in data for “current” control issues only. That is to say that the question was worded in the present tense, that is, “In your relationship with your current/most recent SA husband/partner, would you say it is generally true that he…” with various forms of coercive control following to complete the sentence. A total of 63.4% of the participants stated that they were currently experiencing some type of control with their SA partner or spouse.

#### Emotional Violence

The form of violence most commonly experienced by participants in this survey was emotional violence with 87.5% reporting having ever experienced one of the four forms of emotional abuse—insults, humiliation, intimidation, and threats of violence—listed in the survey.

Insults were the most repeatedly experienced form of emotional violence registered by participants, with 48.6% of participants who had experienced this form of abuse in the previous 12 months saying they had experienced it “many times,” and 43% of participants who had experienced insults historically saying they had experienced it “many times” (52.1% of this latter group having experienced it “a few times”).

#### Physical Violence

In terms of physical violence, 198 (35.3%) of the participants reported ever experiencing physical violence in their relationship with their SA partner, with 17.4% experiencing it currently. Physical violence was measured using six different questions, some of which covered multiple forms of violence, such as the first question which asked participants if their SA husband/partner had ever “slapped you or thrown something at you that could hurt you.”

**Figure 1.**
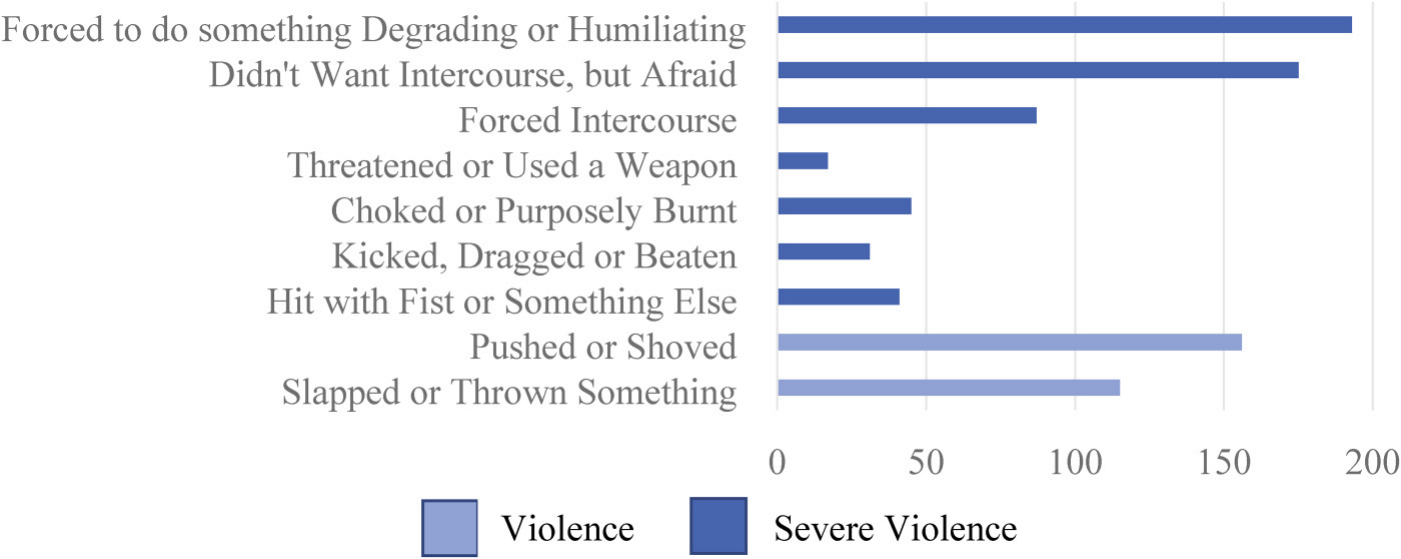
Physical and Sexual Violence.

In the WHO multi-country study (Garcia-Moreno et al., 2005), the researchers distinguish between moderate violence—that is, experiences of being slapped, having an object thrown at one, or pushed/shoved—and severe violence. In total 191 participants (34.2%) had experienced moderate levels of physical violence, while 83 (14.9%) had also (or “only,” in a few cases) experienced severe levels of physical abuse. See Figure 1 for more on the types of moderate and severe violence measured in this study.

#### Sexual Violence

Survey participants were given three “yes or no” questions on the theme of sexual violence. Two hundred thirty-one (41.4%) of them answered “yes” to ever experiencing at least one form of sexual coercion or violence. Figure 1 shows the questions along with number of participants who reported having experienced each type of sexual violence.

Regarding the question on being forced to do something degrading or humiliating, “a few times” was the most chosen frequency option for this question, selected by 59% of the participants who experienced this form of abuse (currently or ever). Fifty-three participants, or 27.5% of those who ever experienced this type of abuse, reported experiencing it “many times” and 13.5% reported experiencing it “once.”

#### Physical and/or Sexual Violence

The percentage of participants in this study who had experienced P/SIPV ever with their SA partner was 57.7%, with 26.3% having experienced it in the last 12 months. By contrast, the WHO, 2013 global estimate for IPV over the course of the entire lifespan was 30.0% (WHO, 2013, p. 16). WHO lifetime prevalence estimates for “high-income” areas of the world—consisting of countries in which most of the study participants (all but four) resided—is 23.2% (2013, p. 17).

The WHO (2013) also distinguishes between normal and severe IPV. The definition for the latter is ever experiencing any form of sexual violence or any of the acts of “severe intimate partner violence.” The percentage of study participants who met the “severe P/SIPV” criteria was 47.5%.

#### Violence Against Children

Of the 492 participants who indicated they had children, 104 (21.1%) answered “yes” to one or both questions on the issue of violence against children. These questions were designed to determine whether a participant's child/ren had ever experienced threats of, or actual physical violence, at the hands of the SA partner/spouse. Of these 104 participants, 102 (98.1%), had suffered some form of violence themselves, with 81.7% having reported experiencing P/SIPV.

Twenty-one percent of those who said their children had “ever” experienced acts of physical violence at the hands of their SA partner/spouse reported this violence was current with the majority of these (70.6%) saying it had occurred “a few times” and the remainder saying it had happened “once.”

### Help Seeking

Three hundred twenty-seven of the 514 participants who had experienced control/violence (63.6%) reported that they had told someone about these experiences. The most chosen confidant was “a counselor/mental health professional” (78.9% of the help-seeking participants) with “a friend” (77.1%) a close second.

**Figure 2.**
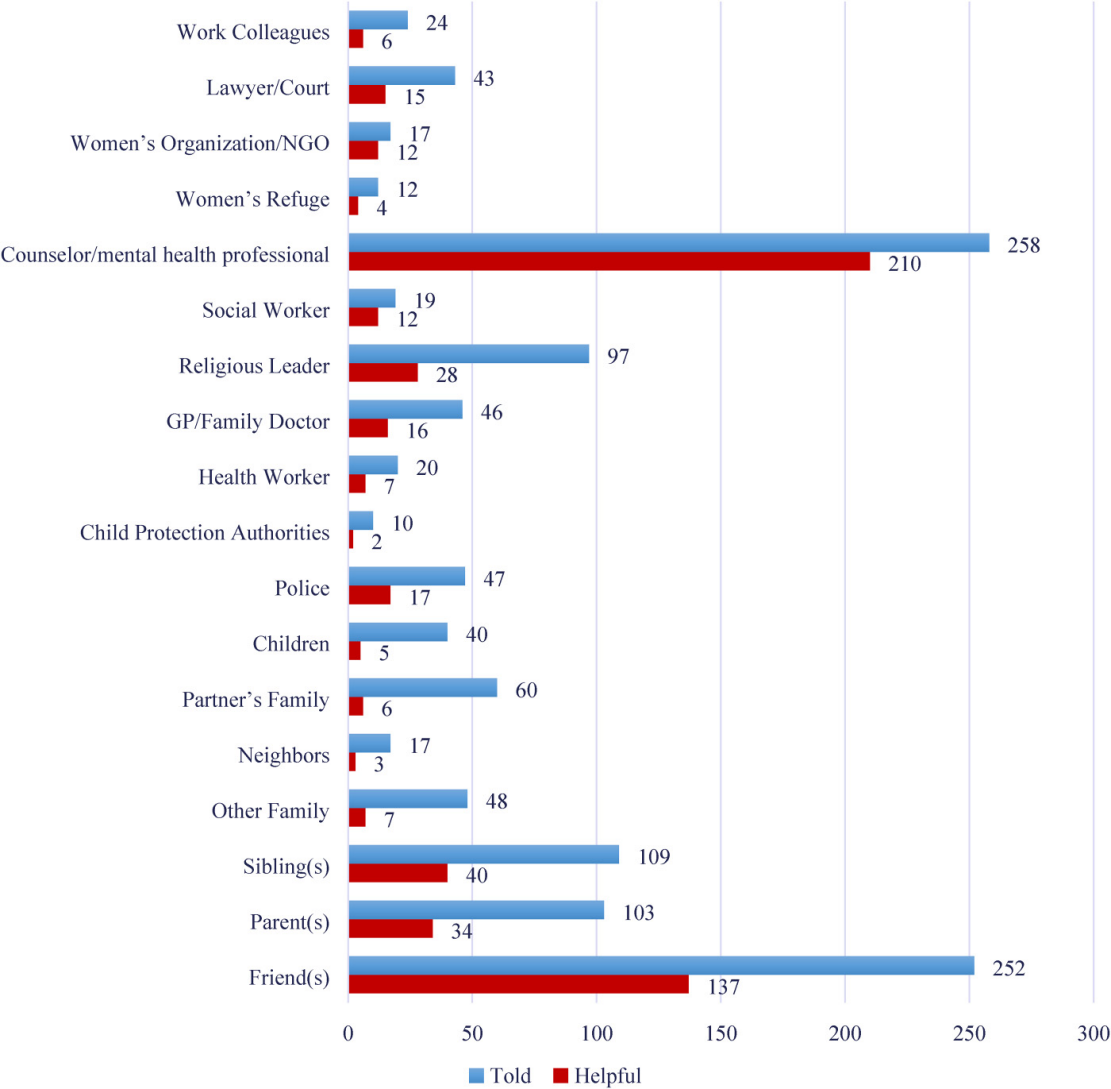
Help-Seeking Behaviors.

Figure 2 shows the number of participants who reported having sought help by telling various people/agencies about the violence. It also indicates which of these people/agencies they told were deemed to have been helpful. For example, 81.4% of participants who spoke up to a counselor/mental health professional found them to be helpful. By contrast, only 54.4% of those who talked to a friend found this helpful. And while fewer women spoke to a women's organization/NGO or a social worker, these were listed as the next most helpful by 70.6% and 63.2% of the relevant participants, respectively. Of the 327 participants who did speak to someone about the abuse, 12.5% said “no one” they spoke to turned out to be helpful.

### Hypothesis Testing

The researchers also tested a hypothesis, based on one of the researcher's observations, and backed up by the literature (Craig et al., 2008; Morgan & Gilchrist, 2010), that more extreme forms of sexual acting out, that is, particularly sex offending behaviors, would be associated with P/SIPV perpetration, and possibly severe P/SIPV perpetration. A second hypothesis they wished to test was that the SA's engagement in therapy, and also his engagement in “quality recovery” (as deemed by the participant), would be mitigating factors for violence.

#### Acting Out Behaviors

As previously mentioned, participants were asked to select which types of behaviors they knew their SA partner's acting out to include. The researchers looked at whether the variables on the SA's acting out behaviors, as reported by participants, could be found to produce any statistically significant associations with the various forms of violence. The hypothesis can be expressed as:

*H_O_*: No relationship exists between the SAs’ engagement in particular acting out behaviors and P/SIPV perpetration in this population.
*H_A_:* A relationship exists between the SA's engagement in particular acting out behaviors and P/SIPV perpetration in this population.

In Chi-square analysis, highly statistically significant associations were found between all illegal forms of acting out (stalking, voyeurism, and exhibitionism—variables which had statistically significant associations with each other) and both P/SIPV and severe P/SIPV. With combination variables for the illegal behaviors and P/SIPV and severe P/SIPV the results were (χ^2^ 12.44, *p* < .001) for P/SIPV and (χ^2^  19.28, *p *< .001) for severe P/SIPV. The Bayes factor supported the alternative hypothesis for P/SIPV, *BF_01_* = 53 (P/SIPV); and strongly supported the alternative hypothesis for severe P/SIPV, *BF_01 _*= 1497. The odds ratio showed that the odds of P/SIPV perpetration by those reported as having these illegal behaviors was 1.99 times higher than for those who were not reported as having them. These odds increased to 2.28 times higher for severe P/SIPV perpetration.

Thus, the alternative hypothesis (*H_A_*) was supported. Table 2 gives a breakdown of all the statistically significant (*p* < .05) and highly statistically significant (*p* < .01) associations between acting out behaviors and experiences of P/SIPV.

#### Recovery Behaviors

The second hypothesis tested was, “the SA's engagement in specialized therapy, or other mental health support, would be a mitigating factor for current violence.” The hypothesis can be expressed as:

*H_O_*: No relationship exists between SAs’ engagement in specialized therapy, or other mental health support, and current physical and sexual violence perpetration in this population.
*H_A_:* A negative relationship exists between SAs’ engagement in specialized therapy, or other mental health support, and current physical and sexual violence perpetration in this population (i.e., the direction of the relationship will be negative, meaning engagement is associated with less violence perpetration).

The alternative hypothesis (*H_A_*) was supported for:

- Seeking specialist support and current sexual violence (χ^2^  9.030, *p* < .003). The Bayes factor supported the alternative hypothesis *BF_01_* = 6.26. The odds ratio showed that the odds of an SA partner perpetrating sexual violence in the last year was 2.60 times higher if he had not engaged in specialist support.
- Seeking any mental health support (including specialized support) and current sexual violence (χ^2^  7.322, *p* < .007). The Bayes factor supported, although not strongly, the alternative hypothesis *BF_01_* = 3.12. The odds ratio showed that the odds of an SA partner perpetrating sexual violence in the last year was 2.24 times higher if he had not sought any type of support.
- Seeking specialist support and current physical violence (χ^2^  4.863, *p* < .027). The Chi-square calculation shows an association, and the Bayes Factor supported this, although not strongly *BF_01 _*= 1.3. However, a look at the odds ratio indicates that the odds of an SA partner perpetrating physical violence in the last year were 1.98 times higher if he had not engaged in specialist support.

The null hypothesis (*H_O_*) cannot be rejected for “seeking (any) mental health support” and “current physical violence.”

## Discussion

The results of the “Prevalence of DV in the Lives of Heterosexual PSAs” study provide insight into the DV experiences of 558 PSAs residing in a variety of high-income countries. This insight is that violence is prevalent, severe, and frequent in these women's lives, with rates running higher than in the general population. Given what past DV researchers have uncovered, these results should, perhaps, not surprise us.

### Lessons From DV Theory

As far back as the 1980s, feminist academics—along with academics from various disciplines such as psychology, sociology, and criminology—were beginning to posit theories about, and research the causes of, DV through the lens of their specific discipline or ideology (Heise, 1998). It was Bonnie Carlson (1984) who first suggested applying the multiple-factor, ecological framework, developed for understanding child abuse (Belsky, 1980), to VAW. Lori Heise (1998) would later refine this model, at the same time expanding it to include findings from international and cross-cultural research. Heise's ecological model would form the basis for what remains the most common paradigm for understanding risk factors associated with IPV and DV (Garcia-Moreno et al., 2012). The ecological model, “proposes that violence is a result of factors operating at four levels: individual, relationship, community and societal” (Garcia-Moreno et al., 2012, p. 3).

It is particularly worth noting that within the “relationship” category of the ecological model both “conflict or dissatisfaction with the relationship” and “man having multiple partners” (Garcia-Moreno et al., 2012, p. 4) are listed as risk factors for violence. If we assume that “multiple partners” means multiple sexual partners, and that the term also applies to virtual partners, then both of these factors associated with IPV were present in the lives of most of the PSAs who participated in this study. Given that the presence of multiple risk factors is known to increase the likelihood of abuse (Fanslow et al., 2016), a high prevalence of DV in PSAs’ lives might well have been anticipated.

### Pornography Studies

In the words of Bridges et al. (2015): “Among academics, pornography has endured as one of the most combative and divisive areas of research, splitting feminist researchers across the academy into warring factions” (p. 1). Indeed, almost as soon as studies began to emerge demonstrating that pornography is associated with actual VAW, or attitudes conducive to violence, “pro-porn” (for want of a better term) academics emerged to refute their arguments. The latter cohort has used observational studies (Fisher et al., 2013) which they claim are more reliable than the lab-based studies (Linz, 1989) and Confluence Model studies (Malamuth et al., 1995) of the “anti-porn” academics. This observational research includes statistics showing the availability and consumption of pornography increasing in certain geographic locations, while rape reported to the authorities declined in those locations within the same period (Kutchinsky, 1991). Critics of these observational studies point out that rape is a vastly under-reported crime making such comparisons unreliable. Moreover, during the recent COVID-19 lockdown period, researchers have demonstrated that pornography viewing increased in the United States (Awan et al., 2021), as did sexual VAW (Jetelina et al., 2020).

Even if we were to believe the pro-porn academics’ assertions about the limitations of laboratory and Confluence Model studies, there are, nevertheless, many other types of studies which demonstrate a direct link between pornography and inegalitarian attitudes and/or actual acts of aggression and VAW. These include real-world quantitative studies (Mikorski & Szymanski, 2017; Russell, 1998), and qualitative studies (DeKeseredy & Hall-Sanchez, 2016; Sun et al., 2017). There are also studies showing that higher pornography use is correlated with a lower likelihood of intervening to prevent sexual assault (Foubert & Bridges, 2017) as well as two meta-analyses demonstrating that the majority of studies point to a link between pornography use and actual acts of verbal, physical and sexual aggression (Malamuth et al., 2000; Wright et al., 2015).

Despite this wealth of various kinds of research demonstrating an association between pornography use and attitudes conducive to violence and/or actual acts of aggression—over 100 on the latter alone, according to Foubert (2017)—the debate between “pro” and “anti” porn academics rages on. A look at the latter's research does, however, seem to back up critics’ claims that the pro-porn cohort tends to rely on a small number of outlying studies, thereby misrepresenting the full body of research available linking pornography and aggression (Malamuth et al., 2000; Wilson, 2020), and that at least some of these outliers appear to employ questionable methodology (Foubert, 2017; Wilson, 2020).

### Internet Pornography Use and Violence

Given the wealth of literature showing an association between pornography use and IPV or IPSV, it was somewhat surprising that the current study did not turn up such an association with internet pornography, even while associations between violence and other forms of sexual acting out—including other forms of pornography such as “sexualized media”—did appear. However, on consulting with one of the original moderators of an online statistic forum (Glen_b, 2021), it was suggested that the fact that 95% of participants’ husbands were said to be using internet pornography might be the reason for the lack of statistical association. “The imbalance in the [porn use/no porn use],” he explains, “is a problem because you need a fairly strong effect to pick anything [i.e., an association] up” (Glen_b, 2021, para 4).

### Physical Versus Sexual Violence

While disturbingly high, experiences of physical violence were noticeably less common in our study's population (35.3%) than sexual violence (41.4%), in contrast with the results of the WHO study (Garcia-Moreno, 2005, p. 27) and other large IPV studies such as the Center for Disease Control's “The National Intimate Partner and Sexual Violence Survey” (Smith et al., 2015). In the latter study 18.3% of participants reported experiencing contact sexual violence with an intimate partner versus 30.6% experiencing physical violence (Smith et al., 2015, p. 8).

One factor that may contribute to our results is that it is difficult to make stark delineations between “sexual” and “physical” violence (Carney & Barner, 2012). For example, if a PSA has experienced being choked during sex, she could conceptualize that as either physical or sexual violence. Thus, it is possible that the higher sexual abuse to physical abuse results in this study are falsely skewed because the measures used did not delineate between the two sufficiently for participants. However, according to Carney & Barner (2012): “because sexual coercion is often nested within a larger pattern of relationship violence, [it] stands as an unreported or underreported correlate to the already underreported phenomena of physical IPV” (p. 290).

This theory is supported by the above example: “choking during sex.” In this survey, it is more likely participants would have registered this as a type of physical assault, rather than an act of sexual violence, given that “choking” is one of the physical violence measures, and there is no measure in the sexual violence category that would readily accommodate reports of such an experience. Thus, if this study's sexual versus physical abuse data is skewed, it is as or more likely that the sexual abuse is underreported in favor of the physical, and not the other way around.

### Emotional Abuse and Control

Emotionally abusive and controlling behaviors by the SA partner were reported to be more common yet—87.5% and 63.4% of participants, respectively, noting these. This pattern is in line with DV behaviors reported in some of the areas of the world in which the study participants resided: including New Zealand (Fanslow & Robinson, 2011), the United States (Carney & Barner, 2012), and the European Union (Goodey, 2017). These studies discuss the fact that coercive control is often a precursor to other forms of violence (Fanslow & Robinson, 2011) and also a behavior that is intertwined with other forms of violence (Carney & Barner, 2012; Goodey, 2017).

### Age and Violence

While one participant aged 18–19 did not report experiencing P/SIPV, participants in their 20s demonstrated a high rate of P/SIPV, for both “ever”—66.7% of 20–24 year-olds; 50.0% of 25–29 year-olds—and “last 12 months”—66.7% of 20–24 year-olds; 43.8% of 25–29 year-olds.

Upon examining the data from their own and others’ IPV studies, the authors of the WHO (2013) “Global and Regional Estimates of Violence against Women” report noted that violence was showing up even in the youngest demographic included in the studies, “suggesting that violence commonly starts early in women's relationships. Prevalence then progressively rises to reach its peak in the age group of 40–44 years” (p. 16). The results of our study told a similar story. Participants in their 20s demonstrated a high rate of current P/SIPV—66.7% of 20–24 year-olds, 43.8% of 25–29 year-olds. Likewise, those in their mid-40s had the highest rates of relationship-long “sexual violence” and combined “sexual and physical violence.” One difference in our study's findings, however, was that the participants over 70 (15 participants), not their 40s, showed the highest rate of relationship-long physical IPV.

### Geographic Location and Violence

As already noted, the vast majority of the participants in our study were residing in the United States at the time the survey was conducted. Looking at the portion of this sample who stated they lived in the United States (444 participants), 57.4% reported experiencing some form of P/SIPV. A CDC IPV study (Smith et al., 2015) found that 25% of American women had experienced some form of IPV over the course of their life. For the purposes of the CDC study, IPV was defined as “contact sexual violence, physical violence, and/or stalking by an intimate partner” (p. 8).

Though our study's sample size for other nations and regions is much smaller than for the United States, it is still worth noting how the P/SIPV prevalence of PSAs in another region compares with IPV prevalence in the broader community of women in that area. A 2014 population study (Goodey, 2017) estimated IPV prevalence for women in the European Union (then including the UK) at 22%. A look at the 42 PSA participants in this study who were residing in the European Union (including the UK) shows that 59.5% reported having ever experienced P/SIPV.

### Help Seeking

One unexpected result that came out of this study was how few participants experiencing violence had told someone about the experiences—63.6% compared with a New Zealand statistic of 76.7% (Fanslow & Robinson, 2010), a United States estimate of “up to 75%” (Robinson et al., 2020, p. 2) and a Canadian statistic of more than 80% (Barrett & St. Pierre, 2011). This result is particularly surprising given that the participant information sheet for this study stated the criteria for inclusion was that women “identify themselves as a PSA and… have sought support specifically for this issue (e.g., from a therapist, coach, PSA support group, religious leader).”

One possible explanation for the lack of DV disclosure is shame. Betrayal is itself associated with shame (Platt et al., 2017), even when there is no co-occurring DV. Moreover, researchers have noted that shame runs high in victims of IPV (Thaggard & Montayre, 2019) and impedes women's help-seeking (Mantler et al., 2021; Thaggard & Montayre, 2019). Another possible reason for the low DV disclosure rates was failure, on the part of formal support, to assess for DV and/or acknowledge the possibility of its presence. This can also discourage victims from disclosing (Todahl et al., 2020), thereby potentially leaving them at risk of harm.

On the topic of help-seeking, there was one other somewhat surprising result: the most commonly chosen single confidant for the participants was a formal support (i.e., a counselor/mental health professional) rather than an informal support person (e.g., a friend). As in other studies (Barret & St. Pierre, 2011; Fanslow & Robinson, 2010), informal supports were, as a class however, disclosed ahead of formal, professional supports, though by a narrower margin (86.2% vs. 85.0%) than in these earlier studies.

### Limitations and Strengths

Key DV studies to date have used some form of random sampling to obtain participants (Ellsberg et al., 2003). Indeed, probability sampling, with its ability to achieve “representativeness” is still held up as “the gold standard” in most general population social science research (Stern et al., 2017). However, in a day of nearly ubiquitous access to the internet, in most societies, and “decline in response rates across traditional modes of data collection” social scientists are “asking how to best adapt to changing times while retaining the quality of the data collected” (Stern et al., 2017, p. 714).

Moreover, for the very specific, and difficult-to-reach population “heterosexual women who self-identify as PSAs and who are seeking/have sought, support for this issue” it was deemed that random sampling was not the best option. Thus, nonprobability methods—specifically restricted self-select sampling and list-based sampling—were used. And while this means that the results, unlike other DV studies, are not broadly generalizable (Fricker, 2017), they do capture the experiences of this moderately large sample at the time of participation. Moreover, it can be argued that these results have a degree of generalizability to nonparticipants who share similar characteristics (Bhandari, 2020).

As for strengths, by surveying an international sample of heterosexual women who identify as PSAs, and who may reflect the characteristics of a broader PSA population, this study has provided a baseline of quantitative data about the worldwide prevalence of DV and IPV in the lives of such women. The anonymous nature of the survey protected the participants’ identities, allowing them to share, without fear of reprisal, their experiences of violence.

Moreover, because the study was designed to closely emulate the WHO multi-country study (Garcia-Moreno et al., 2005)—which has been demonstrated to have high internal consistency (Schraiber et al., 2010)—this study likely attained at least some of the WHO's high standards for quantitative VAW research. Moreover, because of the crossover in design, the data of the current study could more readily be compared to the WHO data (Garcia-Moreno, 2005; WHO, 2013) as well as the data from other internationally recognized VAW studies, such as those by Fanslow and Robinson (2011), Goodey (2017), and Smith et al. (2015). By making these comparisons with general population studies, the magnitude of the DV and P/SIPV problems these PSAs face becomes easier to grasp.

## Conclusions and Recommendations

The primary aim of this study was to quantify how prevalent and frequent various forms of DV have been in the lives of a moderately large cohort of PSAs, and thus, potentially in the lives of other PSAs who share similar characteristics. Since the answer to that question was “it is extremely prevalent and far too frequent,” action clearly must be taken to help ensure the safety of PSAs and address violence perpetration in the relationship. One of the study participants who contacted the researchers had this recommendation for the SA/PSA counseling fields:

It is my hope that through your research, a paradigm shift can be made to address domestic violence within the context of a marriage impacted by sexual addiction. I have experienced and observed that addressing the abuse gets overlooked in light of sexual addiction recovery and marriage restoration. I have gained strength and courage to face this issue in my marriage, but it is not an easy task. People helpers, friends and even trained professional counselors seem to have much to learn in how to deal with these situations. (Participant)

### for PSA Supports

This study revealed that counselors/mental health professionals were the people PSAs were most likely to confide in about the violence, and this disclosure was very often found to be helpful. And while this is cause for celebration within the therapeutic community, the warning previously quoted says there is still room for growth and improvement.

Challenges victims of DV may face when seeking support are lack of assessment for the violence, leading to nondisclosure (Todahl et al., 2020); and victim-blaming therapeutic models, leading to ineffective interventions (Leedom et al., 2019). The latter problem has historically existed for the PSA as well (Steffens & Rennie, 2006). Victim-blaming models, such as co-dependency and self-defeating personality disorder, may not only be unhelpful to PSAs, they may be harmful (Leedom et al., 2019), resulting in increased trauma (Minwalla, 2021) or collusion with the perpetrator of the DV she is experiencing (Mayer, 2017). Either outcome may lead to further disempowerment of the victim and might also jeopardize her health and safety.

Thus, given that this research shows that so many of our PSAs (92% in this study) are also victims of trauma-producing and compounding DV, it is our recommendation that counseling for PSAs—whether they are seeking help for PSA experiences, DV or both—be based on a trauma, and not a co-dependent model. Also, it would seem imperative, given the prevalence of DV amongst PSAs, that PSA counselors screen all clients for DV. Because a higher percentage of younger participants—and those in the early years of the relationship—in this study were currently experiencing sexual violence, younger clients, and those in relationship less than six years, particularly need screening for current DV, especially current sexual violence. To ensure screening is effective, and that therapists know what to do with the results of such evaluations, it is recommended that all PSA counselors receive some training in DV screening, referral, and interventions. For those therapists specializing in VAW, this study suggests that a degree of training in partner-of-sex-addict trauma might be advisable. For both sets of specialists, the researcher highly recommends a deep knowledge of working with complex trauma.

### Consideration of Children

While many people working in the sex addiction/PSB field today make a sharp distinction between sex addiction and sex offending (especially regarding those offenses carrying more severe penalties), this was not always the case (Carnes, 2001; Carnes & Rosenberg, 2014). One of the more obvious ways a child may be victimized by a sex-offending parent is by direct sexual exploitation (Carnes, 2001; Morgan & Gilchrist, 2010). Those of us who work with PSAs will most likely find at some point that we are supporting someone whose child has been/is being victimized by her “sex addict” partner. This itself may require specialist training as well as information on and access to other professional support systems (e.g., child protection services, child/youth counseling services). Moreover, given the overlap between violence against children and their mothers which this study demonstrated, and which is backed up by other research (Fanslow et al., 2016), disclosure of violence against one (e.g., mother) needs to be followed by assessment of victimization of the other (e.g., her children).

### For SA/PSB Counselors

Just as this study has given those counseling PSAs some cause for celebration, so it is with those counseling the person with the PSB. According to this research, counseling—and in particular SA/PSB-specialist counseling—may be associated with lower rates of current P/SIPV. Of course, other factors may be involved in this result, for example, those SAs who present for counseling are also less likely to be currently perpetrating IPV.

In any case, the results of this study would indicate that screening for DV should become standard practice in the work with those with sex addiction/PSB. This is particularly the case where the person with the PSB reports the offending behaviors (stalking, exhibitionism, voyeurism) measured in this study. These particular behaviors demonstrated a strong association with severe P/SIPV. Moreover, the results of this study indicate that it would be advisable for SA/PSB therapists to have training in DV-perpetrator interventions, as well as training in how to protect the partner. Finally, the tenets of SA/PSB therapy models such as the CASRD model (Minwalla, 2021), which “identifies deceptive sexuality as a form of domestic abuse and revises the clinical paradigm of sexual acting-out behaviors” (p. 4) should perhaps take a more prominent place in the SA/PSB counseling field. In this model, it is recommended that “treatment… be informed by a consciousness of an abuse dynamic, an abuser, and a victim(s), not just a sexuality problem” (Minwalla, 2021, p. 29).

### Continued Research

The sex addiction/PSB field is now approximately 40 years old. Nevertheless, ours is the first study designed to verify clinicians’ and researchers’ (Irons & Schneider, 1997) observations that PSAs frequently experience DV in the context of their relationship with their SA partner. Despite the paucity of research, a couple of field leaders have forged ahead and developed models that accounted for abuse in the lives of PSAs (APSATS, 2017; Minwalla, 2021), rather than leave this population waiting for the research to catch up to their reality.

Though direct research into the lives of women who identify as PSAs has been lacking, our study supports the findings of a large body of quantitative and qualitative research into the lives of women who have identified some of the behaviors associated with sex addiction/PSB in their abusive spouse or partner. This literature, along with DV frameworks such as the Ecological Model (Heise, 1998), could have been used to predict that PSAs would be an “at risk” population for IPV, and DV.

The scope for future work in the academic arena is vast. Qualitative studies, in particular, could give us further nuanced insight into the DV, and help-seeking, experiences of PSAs. We hope that this initial study will be the first of many on this population, so that those counseling PSAs and their partners will be better prepared to support victims and, where possible, help perpetrators stop abusive behaviors.
